# Short androgen receptor poly‐glutamine‐promoted endometrial cancer is associated with benzo[a]pyrene‐mediated aryl hydrocarbon receptor activation

**DOI:** 10.1111/jcmm.13291

**Published:** 2017-08-07

**Authors:** Lumin Chen, Bo‐Ying Bao, Wei‐Chun Chang, Jason Yen‐Ping Ho, Bi‐Hua Cheng, Chung‐Lin Wang, Qifeng Tang, Wei‐Chung Cheng, Hui‐Wen Chang, Yao‐Ching Hung, Wen‐Lung Ma

**Affiliations:** ^1^ Graduate Institution of Clinical Medical Science Graduate Institute of BioMedical Sciences Department of Pharmacy China Medical University Taichung Taiwan; ^2^ Sex Hormone Research Center Department of Obstetrics and Gynecology Department of Pathology Research Center for Tumor Medical Science China Medical University/Hospital Taichung Taiwan; ^3^ Department of OBs & GYN BenQ Medical Center Nanjing Medical University Suzhou Jiangsu Province China; ^4^ Department of Nursing Asia University Taichung Taiwan; ^5^ Department of OBs& GYN Chia‐Yi Chang‐Gong Memorial Hospital Chia‐Yi Taiwan; ^6^ Chung‐Jen Jounior College of Nursing, Health Sciences and Management ChiaYi Taiwan

**Keywords:** AR, poly‐Q polymorphism, BaP, AhR, endometrial cancer

## Abstract

The androgen receptor (AR) poly‐glutamine polymorphism (AR‐Q) was reported to play role in endometrial cancer (EMCA) development, yet controversial. Environmental factors interact with genetic variation have been reported in EMCA. Aerosol toxins, polycyclic aromatic hydrocarbon benzo[a]pyrene (BaP), are EMCA facilitators. This report examined the interplay between AR‐Qs and BaP in EMCA. During analysing patient AR‐Q polymorphism and Aryl hydrocarbon Receptor (AhR) expressions, we found overall survival (OS) benefit is ascending with AR‐Q lengths (5‐year OS of 61.3% in Q length <20 and 88% in Q length >23). And AhR is higher expressed in short AR‐Q tumour compared to that in long AR‐Q patient. *In vitro* study found androgen‐response element (ARE) activity descends with AR‐Qs length (Q13 > Q25 > Q35), whereas BaP suppresses ARE activities in EMCA cells. Furthermore, AR‐Q13 (but not AR‐Q25, or ‐35) enhances BaP‐induced dioxin‐responsive element (DRE) activity. Lastly, AR‐Q13 exerts higher colony‐forming capacity than other AR‐Qs, and knock‐down AhR abolished AR‐Q13‐mediated colony numbers. This study demonstrated a possible interaction of gene (AR‐Q polymorphism) and environmental toxins (*e.g*. BaP) to affect cancer progression. A large‐scale epidemiology and public health survey on the interaction of environmental toxin and AR poly‐Q in EMCA is suggested.

## Introduction

EMCA is one of the most common gynaecologic cancers in the Western world 1. According to the National Cancer Institute, there are approximately 55,000 new cases diagnosed and 10,000 EMCA patients died each year in the United States 2. Most EMCA develops after menopause 3—a physiological condition defined by the lack of oestradiol or lower oestrogen levels than present during the fertile period of a woman's life 4. Epidemiological studies have described women with high plasma androgen levels as having an increased risk of developing EMCA 5. This effect is attributed to local aromatization of oestrogens into androgens, which increases the mitogenic activity of tumour cells. Alternatively, androgens can also act on target tissues by interacting with the AR.

The AR gene is located on the X chromosome (q11.2–q12), spans 90 kb, contains 8 exons and encodes for a protein of around 917 amino acids 6. The N‐terminal transactivation domain of the AR protein is indispensable for its genomic activity and is encoded by exon 1 7. This exon contains a CAG repeat (encoding for poly‐glutamine; poly‐Q) that is highly polymorphic in length. It influences the transactivation function of AR 8. A linear increase in poly‐Q length is associated with a progressive decrease in AR activity 9. Several epidemiologic studies have related the poly‐Q polymorphism with a risk of developing some gynaecological tumours such as breast 10 and ovarian 11, 12 cancers although discrepant results have also been reported 13, 14, 15, 16. Some epidemiological studies have observed controversial results between AR poly‐Q length and EMCA risk. This may either promote 17, 18, 19, suppress 17, 20 or have no effect 21. However, there is no explanation for the inconsistency of AR poly‐Qs roles on EMCA.

Environmental toxins have been shown to involve human malignancies including EMCA 22. Those environmental toxins such as polyhalogenated aromatic hydrocarbons (PAHs) are lipophilic xenobiotics, which accumulate in endometrium and are implicated in the aetiology of EMCA 23. The PAH receptor, AhR, is expressed in human normal and malignant endometrium. The physiological role of AhR in endometrial function is the AhR‐mediated regulation of oestrogen‐induced proliferation responses in endometrial epithelial cells 24, 25. However, the AhR role in EMCA is unclear. In addition, not all of the AhR ligand works the same on cells. For example, one report showed a differential effect of benzo(a)pyrene (BaP; air pollutant) *versus* 2,3,7,8‐tetrachlorodibenzo‐p‐dioxin (TCDD; dioxin) on human uterine cell migration 26.

There is increasing interest in studying the interaction of genes and environmental cues in human disease progression including EMCA. This study is particularly interesting to the AR poly‐Q polymorphism and AhR ligands interplay in EMCA. Several *in vitro* studies support this speculation. Björk *et al*. 27 showed that TCDD exerts cell‐dependent AR facilitating activity in short poly‐Q expressing cells. Sanada *et al*. 28 also found that androgen represses AhR‐induced transcriptional activation in human prostate and breast cancer cell lines. Krüger *et al*. 29 reported plastic components activation of AhR and AR *in vitro*.

Here, we conducted a hospital‐based cohort study to associate AR gene poly‐Q polymorphism to disease OS and to relate AhR expression in EMCA patients. Furthermore, we introduced a patient‐related AR poly‐Q cDNAs to co‐treat with AR ligand (5α‐dihydrotestosterone; DHT) or AhR ligands (BaP or TCDD). This shows molecular interactions by measuring ARE or dioxin‐response element (DRE) activities in HEC‐1A EMCA cells. Finally, we knocked down AhR by short‐hairpin RNA to observe colony‐formation capacity to test this hypothesis on the cellular level.

## Materials and methods

### Patient recruitment

Specimens (including blood DNA and paraformaldehyde embedded EMCA tissue) analysed in this study were obtained from patients diagnosed with EMCA from 2003 to 2006 at the China Medical University (Taichung, Taiwan). Patients were identified from a single cohort registered in the Cancer Registry Database of the hospital, and EMCA pathology was classified according to World Health Organization pathology classification. Access to the tissue samples was approved by the Internal Review Board of the China Medical University Hospital (#DMR101‐IRB2‐276). A total of 100 Taiwanese patients with EMCA were recruited, and the patient demographic characteristics are shown in Table 1.

**Table 1:** Demographic and clinicopathological characteristics of patients with endometrial cancer

Characteristic	No. of patients	No. of death	5‐years OS (%)^a^	*P* b
Age at diagnosis, years				
≤53	46	4	83.1	0.281
≥53	54	8	77.8	
BMI, kg/m^2^				
≤26	49	6	81.0	0.564
>26	46	4	79.9	
Histological type				
Endometrioid	87	9	80.0	0.242
Non‐endometrioid	13	3	67.1	
Grade				
1 + 2	70	3	94.4	<0.001
3	27	8	40.8	
Stage				
I + II	66	3	80.8	0.003
III + IV	31	9	68.5	

a The median follow‐up time was 36.4 months.

b *P* value was calculated by the log‐rank test.

### Genotyping of AR poly‐Q polymorphism

Genomic DNA was extracted from peripheral blood using the QIAamp DNA Blood Mini Kit (Qiagen, Hilden, Duetch) and stored at −80°C until the time of study. The method for analysing AR poly‐Q polymorphism has been described in detail previously (rapid and accurate determination of (CAG) repeats in the AR gene using a polymerase chain reaction and automated fragment analysis). Bharaj BS, Vassilikos EJ, Diamandis EP. Clin Biochem. 1999 Jul;32(5):327–32.). The amplified products were separated on the denaturing polyacrylamide gel using an ABI 3730 genetic analyzer and analysed by size using the GeneMapper software (Applied Biosystems CA, USA).

### Immunohistochemistry staining of and scoring of AhR expression on EMCA patient samples

Freshly excised tumour samples (1 × 1 cm) were immediately immersed in 10% PBS‐buffered formaldehyde. The protocols of tissue processing, slicing, de‐waxing, haematoxylin–eosin staining and immunohistochemistry followed previous publications 30, 31. AhR antibody (H‐211, Santa‐Cruz, CA, USA) was used to stain the EMCA slices. The staining intensities were diagnosed and scored following previous publications 32. In brief, the proportion of cells that stained positive for AhR was graded using a five‐point scale (1: <1/100; 2: 1/100 to 1/10; 3: 1/10 to 1/3; 4: 1/3 to 2/3; and 5: >2/3). The intensity of staining was also graded on a five‐point scale (1: none; 2: weak; 3: intermediate; 4: mid‐strong; 5: strong). The proportions and intensity scores were then added together and compared with ARQ polymorphism. The slides were independently examined by two coauthors who were blinded to the polymorphism data.

### Cell lines, stable cell establishment, chemicals and reagents

The human EMCA cell line HEC‐1A was purchased from ATCC (Lot#58087755; ATCC® HTB‐112™; low endogenous AR expressing cells 33) and cultured in McCoy's 5A (HyClone, Ut, USA). The human embryonic kidney cell line HEK293T was cultured in Dulbecco's modified Eagle medium (DMEM) (Gibco, NY, USA) with 10% foetal bovine serum (FBS; Gibco) and 1% penicillin/streptomycin (Invitrogen, CA, USA). The HEK293T cells were obtained from Dr. Yuh‐Pyng Shyr (Center of Molecular Medicine, China Medical University Hospital, Taichung, Taiwan). The cell lines were maintained at 37°C in a humidified atmosphere of 5% CO_2_. The TCDD (2,3,7,8‐tetrachlorodibenzo‐p‐dioxin; CIL) and BaP (BaP; B1760; Sigma‐Aldrich, MO, USA) had final concentrations of 5 nM and 10 μM, respectively, in the individual experiments.

### Construction of pWPXL‐ARQ13, pWPXL‐ARQ25 and pWPXL‐ARQ35 plasmids

The ARQ13 and ARQ35 cDNA were synthesized and cloned (GENEWIZ, NJ, USA) into pWPXL lentiviral‐based vector (Addgene, MA, USA) with the cloning/releasing restriction enzyme Pmel. The sequencing results showed that the cDNA and Q‐lengths are correct (data not shown). The ARQ25 cDNA were amplified from the previously used pBabe‐AR plasmid (Reprod Sci. 2014 Mar; 21(3): 386–394). This was subcloned into the pWPXL vector at the Pmel site.

### Lentiviral‐based gene transduction

The lentiviral production and infection procedures were carried out as reported previously with minor modifications.^34^ Briefly, cells were transfected with the following lentivirus plasmids: psPAX2 packaging plasmid, pMD2G envelope plasmid (Addgene), pWPI‐vector ctrl, or pWPI‐ARQs (‐ARQ13, ‐ARQ25 or ‐ARQ35). Lentiviral plasmids carrying the GFP gene were co‐transfected with psPAX2 and pMD2G into HEK293T cells at a ratio of 3:2:4 with lipofectamine 2000 (Invitrogen) per the manufacturer's instructions. After 6 hrs, the media was replaced with fresh DMEM/10% FBS, and the cells were maintained at 37°C in a humidified incubator in an atmosphere of 5% CO_2_ for 48 hrs. Media containing virus was collected by centrifugation and filtered through a 0.45‐μm filter. Media containing 0.8 mg/ml polybrene (Sigma‐Aldrich) was then added to culture dishes containing 10^6^ HEC‐1A cells. After 16‐hrs infection, the media containing the virus was replaced with fresh DMEM/10% FBS medium, and the cells were maintained at 37°C in a humidified incubator in an atmosphere of 5% CO_2_ for 48 hrs. Infected cells were then collected and analysed. The green fluorescence protein (GFP) + cells were measured with flow cytometry (BD, CA, USA, LSR II Flow Cytometry) to determine infection efficiencies. GFP+ cells with infection efficiencies greater than 85% were subjected to the following experiments.

### Stable transfection of AR poly‐Qs cDNA

The infected HEC‐1A cells were plated onto 10‐cm dishes and treated with puromycin (6 μg/ml) for 3 weeks to form single colonies. The cells were then subcultured, and AR expressions were examined with an immunoblotting assay as described below. After confirming AR poly‐Q expression, the cells were frozen and subjected to cellular and molecular experiments.

### Western blotting assay

Protein extraction and the immunoblot assay were performed as previously described 31. Briefly, cells were washed with 1xPBS and resolved in RIPA buffer (100 mM Tris, 5 mM EDTA, 5% NP40; pH 8.0) with protease inhibitors (1 mM phenyl‐methyl sulphonyl fluoride, 1 μg/ml aprotinin, 1 μg/ml leupeptin). Proteins were separated with SDS‐PAGE and then transferred to PVDF membranes. Blocking of non‐specific binding was accomplished by adding 5% non‐fat milk. After application of primary antibodies (AR, N‐20 Santa Cruz; AhR, Santa Cruz; β‐actin, Santa Cruz, CA, USA), secondary antibodies (1:3000, HRP‐goat‐anti‐mouse and HRP‐goat‐anti‐rabbit) were applied for 1 hr at room temperature. Signals were enhanced using an ECL chemiluminescence kit (Millipore, MA, USA) and detected with ChemiDoc™ XRS+ (Bio‐Rad, CA, USA).

### Gene expression assay and luciferase assay

The assay was performed as previously described 35. Briefly, pGL3‐ARE 36 or pGL3‐DRE 37, 38 and pRL‐TK (thymidine kinase promoter‐driven renilla luciferase plasmid) were transiently cotransfected into cells. After 6 hrs, medium was replaced with fresh medium and 10% CDFBS. Cells were then cultured for 48 hrs with or without DHT (10 nM). After 24 hrs, cells were washed with 1xPBS and then incubated in the presence of 100 μl CCLR (cell culture lysis reagent) (Promega, WI, USA) at room temperature for 30 min. Cell lysates were then placed in a microtube and centrifuged at 12000 g for 5 min. Supernatant (5 μl) was then mixed with 50 μl luciferase assay reagent. Luciferase activity was measured immediately using a luminescence reader (Berthold Detection System FB12 Luminometer) and presented as relative luminescence units.

### Colony‐formation assay

The cells were seeded onto 6‐cm plates (200 cells/dish) with DMEM in 10% CDFBS and incubated for 14 days. In one set of cells, 1000 μl of 4% formaldehyde solution was added to fixed cells, and the cells were allowed to incubate at room temperature for 1 hr**.** Crystal violet cell staining was then performed. After 1 hr, crystal violat was washed from the cell culture dish and cell colonies were photographed.

### Statistics

The associations of patient clinic‐pathologic characteristics with OS and AR poly‐Q lengths were assessed by a log‐rank test and Student's *t*‐test, respectively. The Kaplan–Meier method was used to compare the influence of AR poly‐Q lengths on OS, and the significance was determined using the log‐rank test. Univariate and multivariate analyses to determine the interdependency of AR poly‐Q polymorphisms and clinical risk factors such as age, histological type, grade and stage were carried out using Cox regression 39. Unpaired *t*‐test was used for other experiments, and the standard error of mean (S.E.M.) served as an experimental variation and *P*‐values less than 0.05 were considered to be statistically significant.

## Results

### AR poly‐Q length is negatively associated with EMCA progression and AhR immunostaining intensity

**Figure 1:**
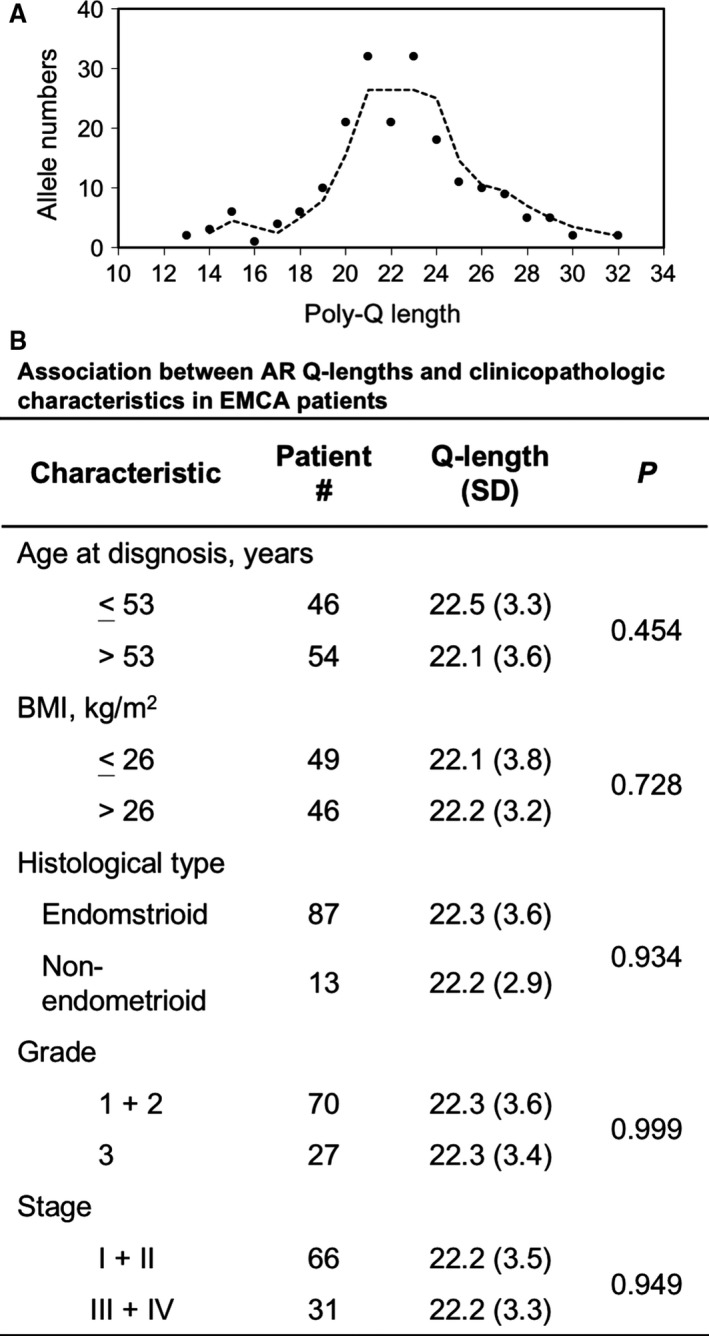
Distribution of the AR poly‐Q lengths and their associations with patient characteristics. (**A**) The frequency distribution of AR poly‐Q lengths in our study population. (**B**) The association between AR poly‐Q lengths and clinical/pathological characteristics in EMCA patients.

The median age at diagnosis was 53 years (range, 32–76 years). There were 12 patients who died from EMCA during the median follow‐up time of 36.4 months. Higher grade and stage of the disease were significantly associated with OS (*P* ≤ 0.003). Five categories were associated with 5‐year OS: age of diagnosis (≤53 or >53 years old), BMI (body mass index; kg/m^2^; ≤26 or >26), histology type (endometroid or non‐endometroid), grade (grade 1 + 2 or 3) and stage (stage I+II or III+IV). The higher grade (*P* < 0.001) and staging (*P* = 0.003) are significantly associated with poorer OS in the study population. The AR poly‐Q length was measured separately on each strand and the frequency distribution of AR poly‐Q lengths in our series. There were 19 different lengths (range, 13–32 repeats). The median number of the repeats was 22. Four of the poly‐Q repeats (20, 21, 22 and 23) had an overall frequency of 53%. The AR poly‐Q length presented a normal distribution in the study population (Fig. 1A). However, AR poly‐Q length was not associated with patient clinicopathologic characteristics (Fig. 1B).

**Figure 2:**
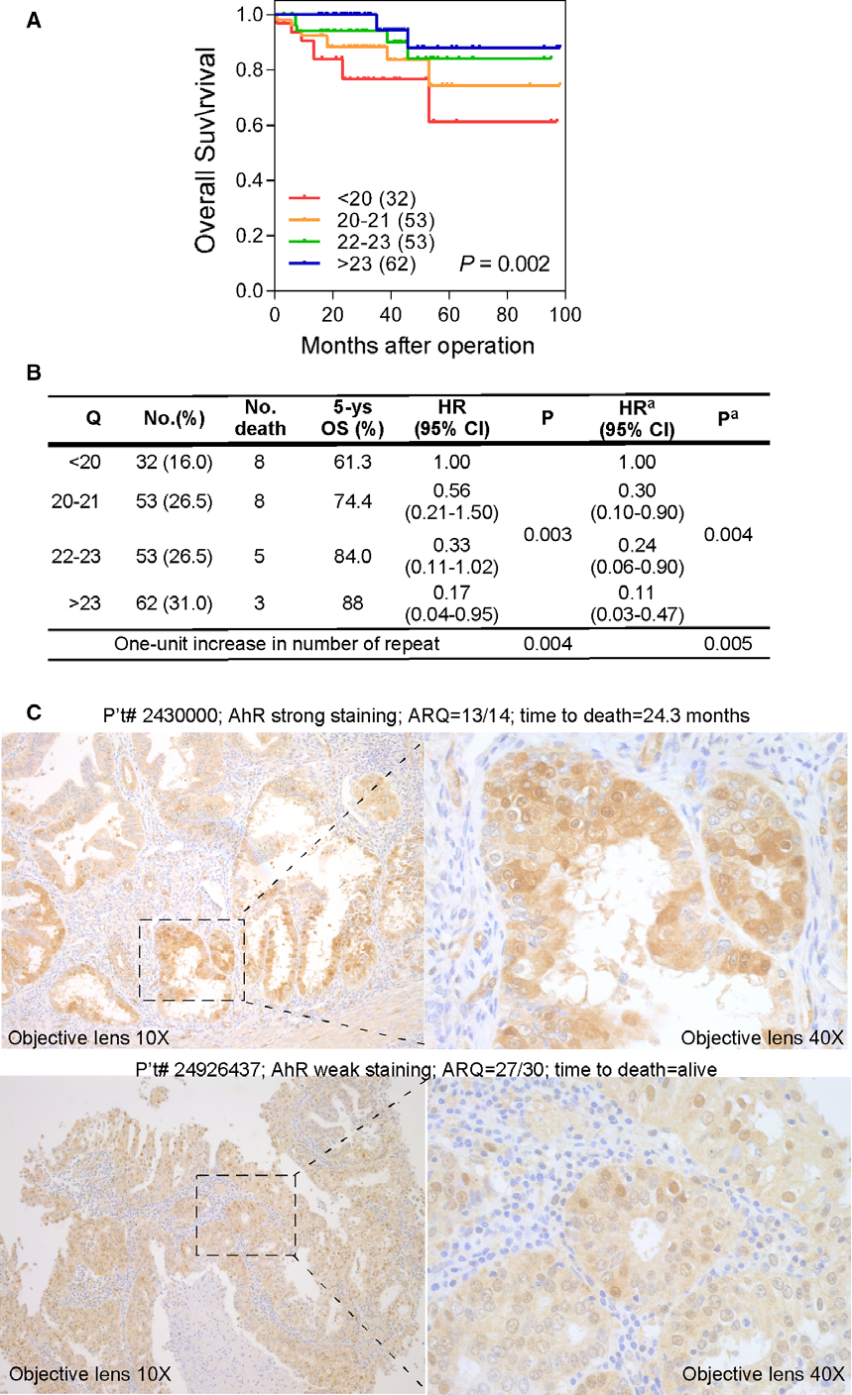
The association of AR poly‐Q length with OS and AhR expression in EMCA patients. (**A**) Kaplan–Meier curves of OS estimated by AR poly‐Q repeat lengths. The repeat lengths were categorized into four groups (<20, 20–21, 22–23 and >23) according to the quartile in our study population. (**B**) Cox regression analysis of AR poly‐Q lengths with OS in EMCA patients. (**C**) AhR immunostainning of EMCA patient (#2430000) with AR Q‐length = 13/14 (two alleles) showed positive staining (upper‐left: 10X; upper‐right: 40× objective lens). The other patient with AR Q‐length = 27/30 (two alleles) showed negative stainings (lower‐left: 10X; lower‐right: 40× objective lens). The inlet of left panels enlarged magnification in the right panels.

We categorized AR poly‐Q lengths into four groups according to the quartile in our series, <20, 20–21, 22–23 and >23. Kaplan–Meier survival curves and log‐rank test revealed that shorter AR poly‐Q lengths were significantly associated with poorer OS (Fig. 2A). A strong gene‐dosage effect on OS was observed when analysed according to a per unit increase in poly‐Q length (hazard ratios (HR) 0.85, 95% confidence interval (CI) 0.76–0.95, P‐trend = 0.004; Fig. 2B). After adjusting for clinical risk factors in the multivariate analysis, the AR poly‐Q polymorphism was further identified as an independent prognostic factor for OS in EMCA patients (Fig. 2B).

We studied AhR expression with immunostainning assay in the patient tumour lesions. We found highs staining intensity in short AR‐Q patient (Fig. 2C; upper panel; patient#243000; Q length 13/14 in two alleles of X‐chromosome) and low staining intensity in long AR‐Q patient (Fig. 2C; lower panel; patient#24926437; Q length 27/30 in two alleles of X‐chromosome). Meanwhile, the short AR‐Q/high AhR patient died at 24.3 month of diagnosis, and the long AR‐Q/low AhR patient survived to the end of study (more than 8 years).

The human data demonstrated that the short AR poly‐Q is strongly associated with EMCA prognosis; this phenomenon likely links to AhR activation or expression. Therefore, the following experiment will examine this possibility of interaction between ARQ and AhR function.

### Patient‐related short poly‐Q AR facilitates BaP‐induced AhR activation, which promotes cancer cell growth

**Figure 3:**
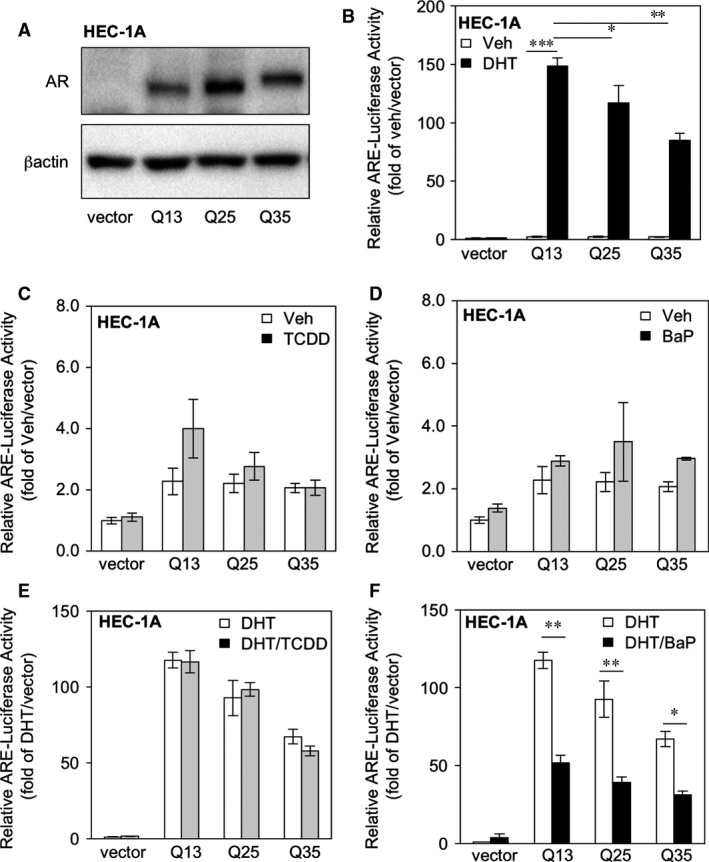
BaP suppresses AR poly‐Q‐related ARE activities in HEC‐1A cells. (**A**) Establishment of AR poly‐Q stable expressing HEC‐1A cells and confirmation by immunoblot assay with anti‐AR antibody. The vector is pWPX plasmid transfectant, and Q13, Q25 and Q35 indicate various AR poly‐Q length cDNA transfectants. Detection of β actin is the loading control. (**B**) ARE luciferase activities measured in the vector as well as Q13, Q25 and Q35 cells treated with or without 1 nM DHT. The reading is the relative luciferase activity (normalized with transfection control pRL‐TK) compared with vehicle (veh)‐treated vector transfectants. (**C**) ARE luciferase activities measured in vector, Q13, Q25 and Q35 cells (in the absence of DHT) to co‐treated with/wo TCDD. The reading is the relative luciferase activity compared with veh‐treated vector transfectants. (**D**) ARE luciferase activities measured in vector, Q13, Q25 and Q35 cells (in the presence of 1 nM DHT) to co‐treated with/wo TCDD. The reading is the relative luciferase activity compared with DHT‐treated vector transfectants. (**E**) ARE luciferase activities measured in vector, Q13, Q25 and Q35 cells (in the absence of DHT) and co‐treated with/wo BaP. The reading is the relative luciferase activity compared with veh‐treated vector transfectants. (**F**) ARE luciferase activities measured in vector, Q13, Q25 and Q35 cells (in the presence of 1 nM DHT) and co‐treated with/wo BaP. The reading is the relative luciferase activity compared with DHT treated vector transfectants. The data were from the mean values of at least five sets of experiments, and S.E.M. was used to show variations. The *P*‐values are less than 0.05 (*), 0.01 (**) or 0.001 (***).

To understand the possible interactions of AR poly‐Qs and BaP‐related molecular events, we first constructed a patient‐related AR poly‐Q cDNA (AR‐Q13, AR‐Q25 and AR‐Q35) that was stably expressed in EMCA HEC‐1A cells (Fig. 3A) and measured ARE‐promoter activity with luciferase assay (Fig. 3B). We found that ARE activity decreases with Q‐length (Q13 > Q25 > Q35), which is comparable with previous conclusions 9. We then treated the cells with or without AR ligand (DHT 1 nM; similar to female androgen level 40, or AhR ligands (BaP, 10 μM; or TCDD, 5 nM). We found that TCDD co‐treatment cannot influence ARE activity—either in the absence (Fig. 3C) or presence (Fig. 3D) of DHT. On the other hand, we found that BaP treatment could not influence basal ARE activity (Fig. 3E). However, BaP could suppress ARE activity in the presence of DHT—particularly in the shorter (AR‐Q13 and AR‐Q25) *versus* the longer (AR‐Q35) transfected cells (Fig. 3F).

**Figure 4:**
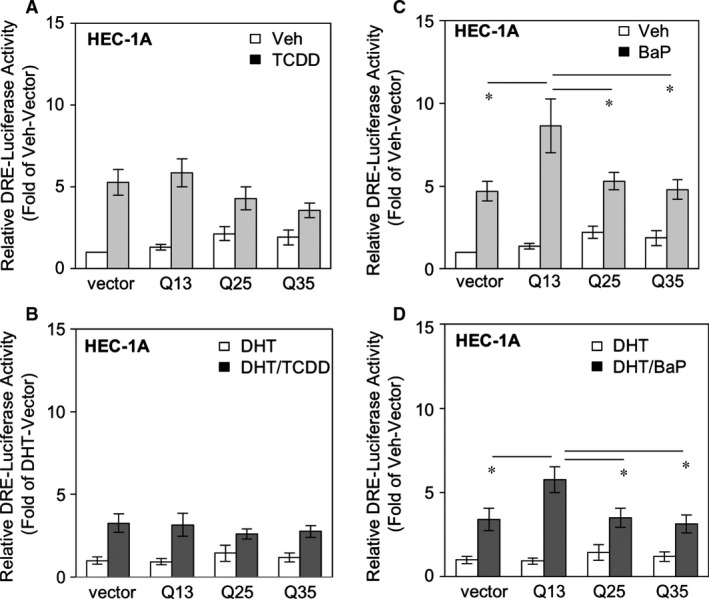
AR poly‐Q13 enhances BaP‐induced DRE activities in HEC‐1A cells. (**A**) DRE luciferase activities measured in vector, Q13, Q25 and Q35 cells (in the absence of DHT) and co‐treated with/wo TCDD. The reading is the relative luciferase activity compared with veh‐treated vector transfectants. (**B**) DRE luciferase activities measured in vector, Q13, Q25 and Q35 cells (in the presence of 1 nM DHT) and co‐treated with/wo TCDD. The reading is the relative luciferase activity compared with DHT‐treated vector transfectants. (**C**) DRE luciferase activities measured in vector, Q13, Q25 and Q35 cells (in the absence of DHT) and co‐treated with/wo BaP. The reading is the relative luciferase activity compared with the veh‐treated vector transfectants. (**D**) DRE luciferase activities measured in vector, Q13, Q25 and Q35 cells (in the presence of 1 nM DHT) and co‐treated with/wo BaP. The reading is the relative luciferase activity compared with DHT‐treated vector transfectants. The data are from the mean values of at least five sets of experiments, and S.E.M. was used to show variations. The *P*‐values less than 0.05 (*), 0.01 (**) and 0.001 (***) are shown.

Our goal was to test the AR *versus* AhR interactions, and we measured DRE in AR‐Qs‐transfected cells. Both TCDD and BaP can induce similar inductions of DRE activities in HEC‐1A cells (Fig. 4). We found either the AR‐Qs themselves (Fig. 4A and B) in the absence (Fig. 4A) or presence of (Fig. 4B) DHT treatment cannot alter TCDD‐induced DRE activity. However, AR‐Q13 can enhance DRE activity in spite of the DHT treatments (Fig. 4C and D). These data indicate that short AR‐Q facilitates BaP‐induced AhR activation, which might be involved in EMCA progression.

**Figure 5:**
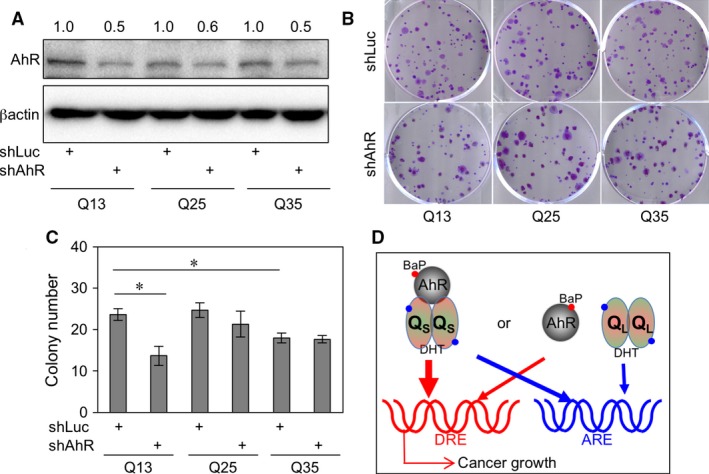
Knock‐down AhR in decreases the colony‐forming numbers in AR poly‐Q13 transfectant of HEC‐1A cells. (**A**) AhR shRNA knock‐down expression in AR poly‐Qs transfectants. The shRNA specific to luciferase (shLuc) or AhR (shAhR) lentiviral‐based gene transduction was delivered into ARQ13, ‐Q25 and ‐Q35 HEC‐1A cells. The knock‐down efficiencies were comparisons within shLuc *versus* shAhR on each ARQs. This is shown on the band. The expression of β‐actin is the loading control. (**B**) Representative image of cell colonies on the 6‐well plates. The shLuc *versus* shAhR‐infected HEC‐1A (AR‐Q13, ‐Q25, ‐Q35) cells were observed. (**C**) Quantitation of **B** experiments and plotted on the bar graph. The colony number is gradually reduced with Q‐length (Q13 = Q25 > Q35). The shAhR suppressed AR‐Q13 promoted the colony number, but not other AR‐Qs. (**D**) The AR‐Qs *versus* BaP‐AhR interaction model of EMCA. Q_S_ = short AR‐Q; Q_L_ = long AR‐Q. The DRE is ‘dioxin‐response element’, and ARE is ‘androgen‐response element’.

To provide cellular level evidence of AR‐Qs and AhR interactions, we introduced AhR knockdown in AR‐Qs transfected HEC‐1A cells and measured HEC‐1A cancer cell growth with colony‐formation assay. As shown in Figure 5A, AhR shRNA could reduce AhR expressions to around 50%. Moreover, the colony numbers in AR‐Q13 transfected cells are more than those in Q25 and Q35 transfected cells (Fig. 5B upper panel; and Fig. 5C, lane 1, 3 > lane 5). Finally, knockdown of AhR in the AR poly‐Q13 could suppress colony number, but not Q25 and Q35 cells. Together, these data suggested that AR poly‐Q13 could facilitate cell growth; reduced AhR could abolish AR‐Q13‐enhanced cell growth.

## Discussion

The multiple‐hit theory suggests that various cancer driver genes promote carcinogenesis; cancer progression is a cancer hallmark 41. This study describes a potential interaction of environmental toxins and gene polymorphism to affect cancer progression. We found that short AR‐Q is an EMCA progression promoter. This mechanism can be separated into two episodes: (*i*) with the calibre of turn on ARE, a short AR‐Q exerts itself better than the AR‐activating capacity (Fig. 5D; blue line on the left‐hand side of cartoon). (*ii*) Short AR‐Q synergizes BaP‐AhR activation to activate target genes containing DRE (Fig. 5D; red line on the left‐hand side of cartoon). On the contrary, the long AR‐Q cannot interact with BaP‐AhR activation; therefore, they are two independent systems in the cancer cells with less gene regulation ability (Fig. 5D; right‐hand side of cartoon). The conclusion of this report can be discussed and might benefit the gynaecological cancer field in the following aspects.

### Short AR‐Q promotes EMCA progression

The AR poly‐Q polymorphism roles in EMCA have been studied since 2000. There are inconsistencies in the literature, and the most controversial part is in the risks of EMCA occurrence. For example, Sasaki *et al*. (2000; West coast US; ~30 patients) 42 and Yaron *et al*. (2001; Israel; ~600 patients) 18 found that short AR‐Q might be associated with EMCA carcinogenesis. On the contrary, a finding by the same group (Sasaki *et al*. 2003; Japan; ~300 patients) 17 found that AR‐Q is longer in EMCA lesions, which means that long AR‐Q might suppress malignant EMCA development. Rodríguez *et al*. (2006; Spain; ~200 patients) 43 conducted a larger epidemiological study that supported this conclusion. However, McGrath *et al*. (2006; US; Caucasian; ~500 patients) 19 and Yang *et al*. (2009; Poland, Caucasian; ~200 patients) 21 published results showing no correlation of AR‐Q with EMCA risks. Finally, the most recent paper published by Ashton *et al*. (2010; Australian Caucasian; ~200 patients) 20 found that short AR‐Q promotes EMCA risk.

In reviewing the related studies, we cannot find particular factors that are involved in the data discrepancies, for example ethnicity, location and study population, that might cause these data variations. Recently, there are several trials to pursue AR roles in EMCA, but from expression angle. Unfortunately, those reports still controversy that AR expressions could either be promoter 44 or be suppressor 45 to EMCA development. Even though the conclusions varied, our study provides important insight in addition to carcinogenesis. We showed that shorter AR‐Q results in poorer prognosis. This conclusion would direct future studies to not only focus on the AR‐Q roles in normal endometrium transformation, but also to EMCA cancer growth or other cellular alterations that are related to cancer prognosis.

### Environmental toxins and gene interaction in cancer progression

AR can interact with AhR to alter cancer cell behaviour. For example, Ghotbaddini *et al*. 46 found that TCDD could alter AR activity differentially in androgen sensitive or insensitive prostate cancer cells. Furthermore, AR‐Q polymorphisms interact with AhR to influence cancers. For example, Björk *et al*. 27 found that TCDD selectively affects prostate cancer cell AR action, which might involve refractory cancer progression. Although those papers discuss AR and AhR interactions, they also presented those data in very specific condition and selected cell lines.

In this report, we found treatments of DHT on EMCA cells showed an interaction of AR‐AhR signalling to affect EMCA progression. However, there is also one possibility of androgen interaction with oestrogen receptors (ERs) to affect AhR function, therefore interferes AhR activation and ultimately EMCA progression. For example, Steckelbroeck *et al*. 47 discovered that aldo‐keto reductases (AKRs) exert the 3beta‐hydroxysteroid dehydrogenase activities to convert DHT to testosterone in test tube. And published by the same group, Penning *et al*. 48 found DHT could convert to oestradiol through AKR1C2 in the prostate. Therefore, examination of DHT‐AR polymorphism through activation of ERs‐AhR signalling might contain some interests to better understand gene *versus* environmental toxin interaction, particular in EMCA patients. From this information, one can image the complexity of environmental toxins and gene polymorphism. To the best of our knowledge, there are no epidemiological studies regarding the environmental toxins of AR gene polymorphism in association with endometrial malignancy. Although the environmental toxin and gene interaction is extremely complex, this report details evidence that BaP‐AhR action might interact with short AR‐Q to affect EMCA progression.

## Conclusion

In this report, we show that shorter AR‐Q has poorer EMCA prognosis. This phenomenon is reversely correlated with AhR expression. We also demonstrate that short AR‐Q facilitates BaP‐mediated AhR activation, which might explain the inconsistencies of AR‐Qs in EMCA development. Therefore, it is suggested that large‐scale epidemiological survey on AR‐Q polymorphism, genome‐wide screening on gene mutation, detecting tumour abundance of environmental toxins and the initiation of public health surveys on EMCA patients are important. Future in‐depth studies would help explain the aetiology, prevention and precision medicine needs for EMCA.
